# The Safety of Long-Term Proton Pump Inhibitor Use on Cardiovascular Health: A Meta-Analysis

**DOI:** 10.3390/jcm11144096

**Published:** 2022-07-15

**Authors:** Dalel Jeridi, Anna Pellat, Claire Ginestet, Antoine Assaf, Rachel Hallit, Felix Corre, Romain Coriat

**Affiliations:** 1 Department of Gastroenterology, Cochin Hospital, AP-HP Center, 27 Rue du Faubourg Saint-Jacques, 75014 Paris, France; anna.pellat@aphp.fr (A.P.); claire.ginestet@aphp.fr (C.G.); antoine.assaf@aphp.fr (A.A.); rachel.hallit@aphp.fr (R.H.); felix.corre@aphp.fr (F.C.); romain.coriat@aphp.fr (R.C.); 2 UFR de Médecine, Faculté de Santé, Université de Paris, 75006 Paris, France

**Keywords:** proton pump inhibitors, heart disease risk factors, long term adverse effects, drug-related side effects and adverse reactions, meta-analysis as topic

## Abstract

Introduction: Proton pump inhibitors (PPIs) are one of the most prescribed classes of drugs worldwide as a first-line treatment of acid-related disorders. Although adverse effects are rare and rapidly reversible after a short exposure, concerns have been recently raised about a greater toxicity on cardiovascular health after a longer exposure, especially when combined with clopidogrel. We aimed to evaluate the safety of long-term PPI use on cardiovascular health in patients with known atheromatous cardiovascular disease. Methods: A literature search was conducted in the PubMed, Embase, and Cochrane Library databases and grey literature in April 2022. Articles published between 2014 and 2022 were considered relevant if they were designed as randomized controlled trials (RCTs) that included post hoc analyses or prospective observational studies and if they investigated clinical cardiovascular outcomes associated with PPI use for 6 months or more in patients suffering from cardiovascular disease requiring antiplatelet agent therapy and/or coronary angioplasty. Statistical analyses were performed using RevMan 5.4 software (Computer program, the Cochrane Collaboration, 2020, London, UK). The risk of bias was assessed using the Cochrane risk-of-bias tool for the RCTs and the Newcastle–Ottawa scale for the observational studies. Results: A total of 10 full-text articles involving 53,302 patients were included. Substantial heterogeneity was found among the 10 included studies. The primary analysis showed no significant differences between the PPI group and the control group for the risks of major adverse cardiovascular events (MACEs), all-cause death (ACD), or target vessel revascularization (TVR) using a random-effects model (OR 1.15, 95% CI 0.98–1.35, *p* = 0.08, I^2^ = 73%; OR 1.24, 95% CI 0.94–1.65, *p* = 0.13, I^2^ = 63%; and OR 1.19, 95% CI 0.76–1.87, *p* = 0.45, I^2^ = 61%, respectively). The primary analysis yielded similar results for the risks of myocardial infarction (MI), stroke, and cardiovascular death (CVD) using a fixed-effects model (OR 0.98, 95% CI 0.88–1.09, *p* = 0.66, I^2^ = 0%; OR 1.02, 95% CI 0.90–1.17, *p* = 0.73, I^2^ = 0%; and OR 1.04, 95% CI 0.94–1.16, *p* = 0.44, I^2^ = 35%, respectively). Likewise, a subgroup analysis based on eight randomized controlled trials failed to identify any association between PPI use and the risks of MACEs, MI, stroke, TVR, ACD, or CVD using a fixed-effects model (overall pooled OR 1.01, 95% CI 0.96–1.06; *p* = 0.66; I^2^ = 0%). The pulled data from the two included observational studies (OS) demonstrated a significantly increased risk of MACEs in the PPI group (OR 1.42, 95% CI [1.29–1.57], *p* <0.001; I^2^ = 0%). In another subgroup analysis, no evidence of an increased risk of adverse cardiovascular events in the co-therapy PPI/clopidogrel versus clopidogrel alone groups was found with the exception of the risk of ACD (OR 1.50, 95% CI 1.23–1.82, *p* = 0.001, I^2^ = 0%). Nevertheless, after performing a sensitivity analysis reaching heterogeneity I^2^ = 0%, the co-prescription of PPIs and clopidogrel was at increased risk of MACEs (*p* < 0.001), CVD (*p* = 0.008), and TVR (*p* < 0.001) but remained statistically non-significant for the risk of MI (*p* = 0.11). Conclusions: The overall results of this meta-analysis showed that long-term PPI use was not associated with an increased risk of adverse cardiovascular events. However, inconsistent results were found for combined PPI/clopidogrel therapy. These results should be considered with caution in light of the significant heterogeneity, the limited number of included studies, and the lack of adjustment for potential confounders.

## 1. Introduction

Proton pump inhibitors (PPIs) are one of the most prescribed classes of drugs worldwide [1]. This phenomenon is largely due to their effectiveness in the management of acid-related diseases such as gastroesophageal reflux disease (GERD), peptic ulcer, gastrointestinal bleeding, and *Helicobacter pylori* infection and the prevention of gastric ulcers in patients on aspirin or non-steroidal anti-inflammatory drugs [2]. Presumed safe, PPIs have been available over the counter since 2003, and previous data reported a significant amount of off-label PPI use, with up to 65% of prescriptions having no appropriate indication in the United States [3]. Omeprazole alone was dispensed more than 70 million times in 2016 [3], and PPIs account for over $10 billion in health care costs, with a global cost exceeding $25 billion per year [4]. Moreover, rebound acid hypersecretion may occur after stopping PPI therapy, leading to the recurrence of gastric symptoms and thus to drug dependency [5]. This PPI overuse raises concerns about the potential risks it could cause, especially in the elderly affected by multiple comorbidities and taking multiple medications [6]. Indeed, recent studies, mostly observational studies, reported various adverse events related to long-term PPI therapy, including the risks of cardiovascular diseases [7], fractures, pneumonia, *Clostridium difficile* infection, impaired absorption of micronutrients, kidney disease, dementia, gastric neoplasia [4,8], and drug-to-drug interactions [3]. Regarding the cardiovascular risk, the concomitant use of clopidogrel and PPIs has been specifically investigated in several studies as clopidogrel and PPIs are both metabolized by the cytochrome P450 isoenzyme 2C19, leading to drug–drug interaction due to competition at the binding site [9].

In this meta-analysis, we aim to evaluate the association between long-term PPI use (defined as exposure ≥ 6 months) and the risk of adverse cardiovascular events in patients with known atheromatous cardiovascular disease using studies with evidence levels I or II according to the evidence-based clinical practice guidelines [10]. The primary endpoint was the overall safety of PPIs. The secondary endpoints were defined as the safety of combined PPI/clopidogrel therapy and the overall safety of PPIs according to study design.

## 2. Material and Methods

### 2.1. Protocol

This systematic review was conducted in compliance with the Cochrane Handbook for Systematic Reviews of Interventions [11] and PRISMA [12] guidelines.

### 2.2. Search Strategy

The literature search was conducted in PubMed, EMBASE, and the Cochrane Library in April 2022. In order to reduce publication bias, we also conducted a search of the grey literature through the Data Archiving and Networked Services and Grey Literature Report databases. The following keywords: (“proton pump inhibitor” OR “proton pump inhibitors” OR “PPIs”) AND (“cardiovascular disease” OR “anti-platelet therapy” OR “clopidogrel” OR “aspirin”) AND (“adverse effect” OR “adverse drug reaction” OR “risk”) were searched. Detailed search terms and combinations used for the literature search are available in online Supplementary Table S1. For the grey literature, we only used the keyword “proton pump inhibitors”. Hand searching of references lists was performed to find any additional appropriate article.

### 2.3. Study Selection Criteria

We limited the searches to articles published from January 2014 to April 2022 written in English or French. We selected randomized controlled trials (RCTs) including post hoc analyses and prospective observational studies reported as full text and published by highly influential journals according to the eigenfactor metrics [13].

Articles were included if patients were aged 18 years or older with atheromatous cardiovascular disease at baseline; the experimental intervention was PPI use for 6 months or longer; PPI use was compared with another PPI (established in the study protocol as not at risk of cardiovascular events), another antacid (established in the study protocol as not at risk of cardiovascular events); placebo treatment; or no treatment. All PPIs were assessed as one drug class considering that all PPIs were sufficiently similar to be combined relevantly as one interventional group. Articles were excluded if the study was designed as a retrospective study or a case–control study; the study involved the general population, an isolated case, pediatric population, or animals; or the study consisted of a meta-analysis or a systematic review (references lists were screened to provide additional citations).

The clinical endpoints were major adverse cardiovascular events (MACEs), myocardial infarction, stroke, target vessel revascularization (TVR), cardiovascular death (CVD), and all-cause death (ACD). The MACEs included cardiovascular death, myocardial infarction, and/or stroke, and/or TVR.

### 2.4. Data Extraction

Data extraction was performed by one reviewer (DJ), and the correctness of the extracted data was verified multiple times. In cases of uncertainty, a second reviewer could be requested. Study characteristics and patients’ characteristics at baseline were collected. The following data were extracted for each included study: study design, first author, year of publication, number of centers, total duration of follow-up, number of patients, mean age, gender, percentage of gender, intervention, comparison, concomitant medications (aspirin, clopidogrel), comorbidities (hypertension, dyslipidemia, overweight/obesity according to body mass index, prior myocardial infarction, prior stroke, smoking status as a major risk factor for cardiovascular diseases), and primary and secondary endpoints in accordance with the clinical endpoints of our meta-analysis (major adverse cardiovascular events, myocardial infarction, stroke, target vessel revascularization, cardiovascular death, all-cause death) (Table 1).

### 2.5. Statistical Analysis

Meta-analysis was performed by calculating the pooled odd ratios with 95% confidence intervals (CI) using Revman 5.4 software. Study results were considered statistically significant for *p*-value < 0.05 and CI excluding 1. Heterogeneity among included studies was assessed using the I^2^ statistic and was considered low if I^2^ < 50% and high if I^2^ ≥ 50% [22]. The pooled effect size was estimated using a fixed-effects model for I^2^ < 50%, while a random effects model was used for I^2^ ≥ 50%. We conducted subgroup analyses to assess the influence of concurrent use of clopidogrel and PPIs on cardiovascular adverse events, as well as the influence of study design on the results obtained. We also performed a post hoc subgroup analysis that investigated the potential adverse effects related to the specific PPI. Sensitivity analyses were also performed to determine the impact of heterogeneity on the original results. Funnel plots were used to investigate potential publication bias.

## 3. Results

### 3.1. Study Selection

**Figure 1:**
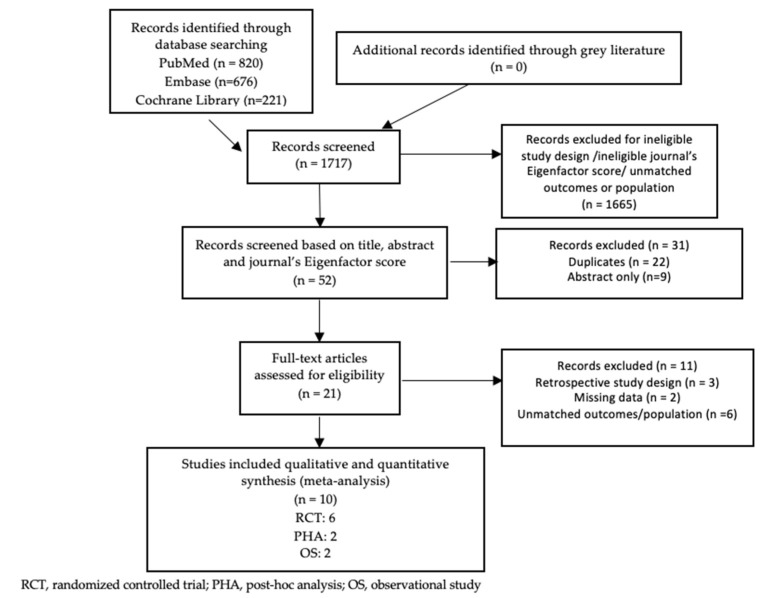
The PRISMA flow diagram for the study selection.

The initial search identified 1717 relevant studies. After title and abstract screening, 1665 studies were excluded. Of the 52 studies remaining, all retrieved from PubMed (*n* = 29), Embase (*n* = 20), and the Cochrane Library (*n* = 3), 22 duplicates and 9 articles only available as abstracts were excluded. Of the 21 articles selected for full-text review, 10 studies met all eligibility criteria and were therefore included in this meta-analysis. Figure 1 shows the PRISMA flow diagram for the study selection.

### 3.2. Study Characteristics

The general study and patient characteristics at baseline are summarized in Table 1. The pooled analysis included a total of 53,302 patients (18,495 patients in the PPI group and 34,807 patients in the control group). The average age was 64.5 years. Both genders were included in all studies, with an average female rate of 29.9%.

Our selected studies numbered eight RCTs [9,14,15,16,17,18,19,23] (including two post hoc analyses [15,16]) and two prospective observational studies [20,21]. Of the ten included studies, two were exclusively conducted in the United States [20,23], two exclusively in China [9,14], one in Italy [16], and five in multiple countries [15,17,18,19,21]. A total of seven studies [9,14,15,16,19,20,21] assessed the cardiovascular risk specifically associated with the concomitant use of PPIs and clopidogrel. The different PPIs used in the intervention groups were lansoprazole [9], omeprazole [14,19,23], esomeprazole [18], pantoprazole [17], and multiple PPIs in two studies [15,16]; this feature was not reported in two studies [20,21]. The control group included patients taking placebo, pantoprazole (considering no affinity between pantoprazole and clopidogrel in this particular study [14]), or no PPI. Patients receiving PPIs were more likely to be older, with a greater prevalence of comorbidities (hypertension, diabetes mellitus, dyslipidemia, and history of stroke and/or myocardial infarction) and polypharmacy (mainly antiplatelet therapy, anticoagulant agents, antihypertensive agents, lipid-lowering drugs, and hypoglycemic agents).

### 3.3. Study Quality Assessment

**Figure 2:**
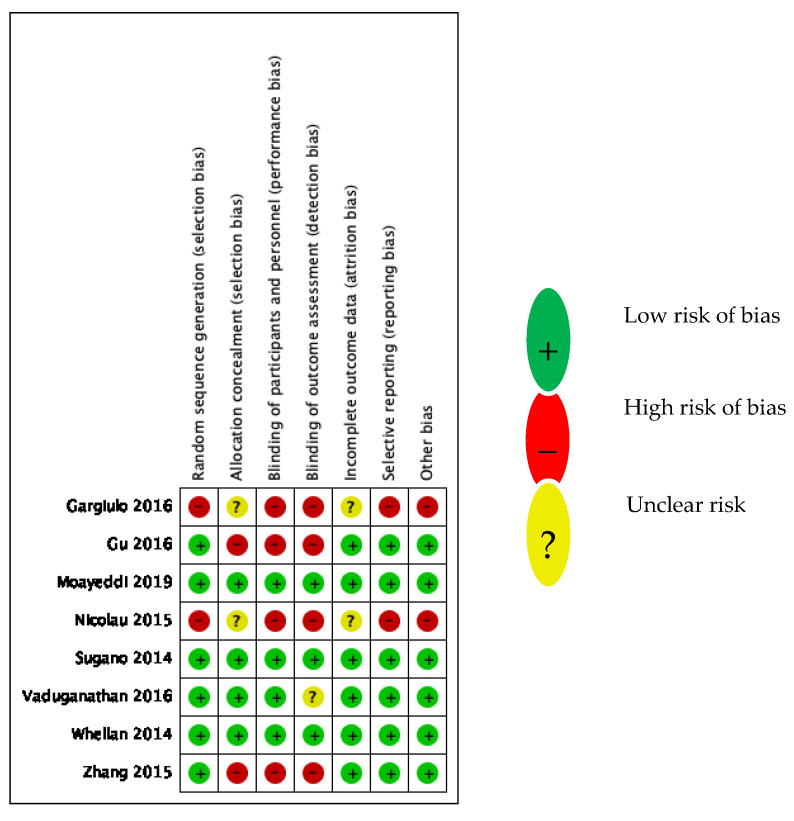
Risk-of-bias summary for the RCTs. Zhang 2015 [9]; Gu 2016 [14]; Nicolau 2015 [15]; Gargiulo 2016 [16]; Moayeddi 2019 [17]; Sugano 2014 [18]; Vaduganathan 2016 [19]; Whellan 2014 [23].

The included studies were of moderate to high methodological quality, except for the PHA, which evaluated the RCTs of low methodological quality, as expected (Table 1). The methodological quality assessment was based upon the Cochrane risk-of-bias [25] assessment tool for the included RCTs and post hoc analyses (Figure 2) and the Newcastle–Ottawa scale [26] (NOS) for the observational studies (Table 2). The Cochrane risk-of-bias tool covers six domains of bias: selection bias, performance bias, detection, attrition bias, reporting bias, and other. Each domain can be assessed as low risk, unclear risk, or high risk. We attributed 0 points, 0.5 point, and 1 point for each domain considered as low risk, unclear risk, and high risk, respectively (Table 1). Three RCTs showed no risk of bias, three RCTs showed one to three risks of bias (selection, performance, and detection), and the two post hoc analyses demonstrated a high risk of bias in all domains except for the risks of attrition and selection bias, which remained unclear (Figure 2). The NOS evaluates the risk of bias in three domains: selection, comparability, and outcome. A score of 6 to 9 was regarded as good, 3 to 5 as fair, and 0 to 2 as poor, according to the NOS interpretation established by Wells et al. [26]. The two included observational studies reached scores of 7 and 8 (Table 2).

### 3.4. Primary Analysis

**Figure 3:**
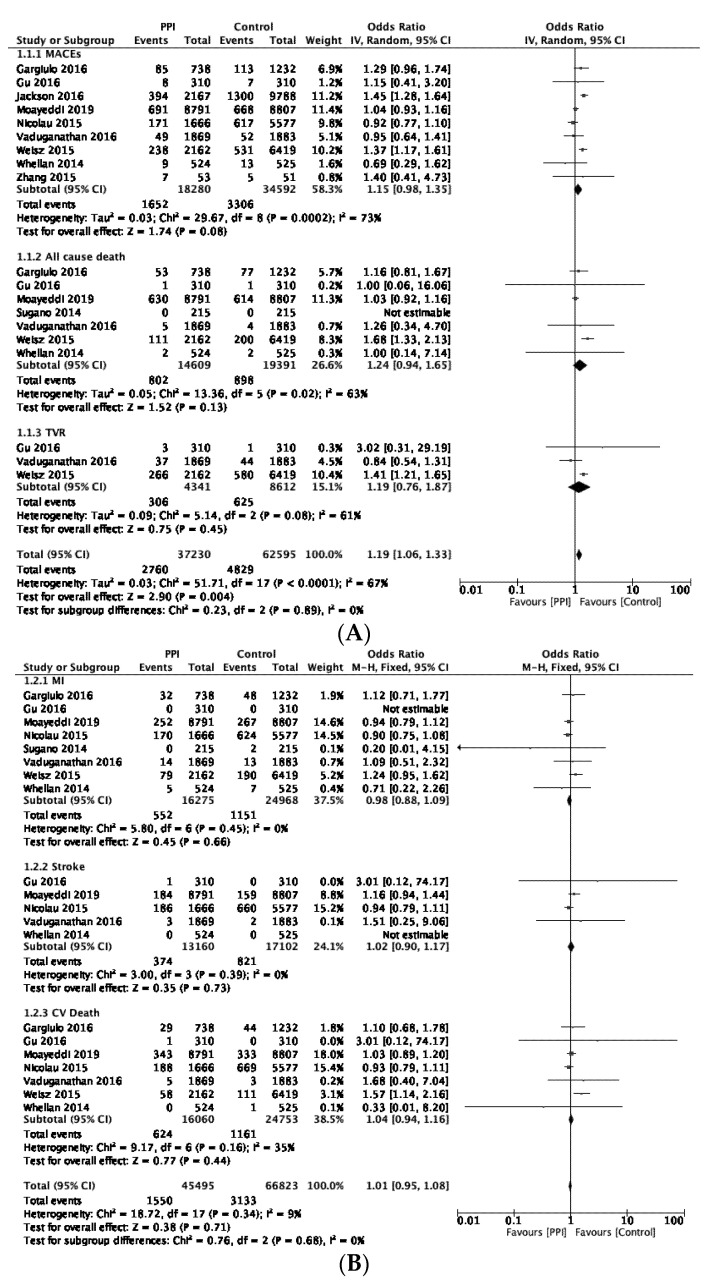
(**A**) Forest plot illustrating the cardiovascular risk associated with long-term PPI use using a random-effects model. (**B**) Forest plot illustrating the cardiovascular risk associated with long-term PPI use using a fixed-effects model. Zhang 2015 [9]; Gu 2016 [14]; Nicolau 2015 [15]; Gargiulo 2016 [16]; Moayeddi 2019 [17]; Sugano 2014 [18]; Vaduganathan 2016 [19]; Whellan 2014 [23]; Jackson 2016 [20]; Weisz 2015 [21].

The results of the primary analysis are presented in Figure 3. We found no significant differences between the PPI group and the control group for the risks of MACEs, ACD, or TVR using a random-effects model (OR 1.15, 95% CI 0.98–1.35, *p* = 0.08, I^2^ = 73%; OR 1.24, 95% CI 0.94–1.65, *p* = 0.13, I^2^ = 63%; and OR 1.19, 95% CI 0.76–1.87, *p* = 0.45, I^2^ = 61%, respectively; Figure 3A). Similar results were found for the risk of myocardial infarction MI, stroke, and CVD using a fixed-effects model (OR 0.98, 95% CI 0.88–1.09, *p* = 0.66, I^2^ = 0%; OR 1.02, 95% CI 0.90–1.17, *p* = 0.73, I^2^ = 0%; and OR 1.04, 95% CI 0.94–1.16, *p* = 0.44, I^2^ = 35%, respectively; Figure 3B).

### 3.5. Subgroup Analysis

**Figure 4:**
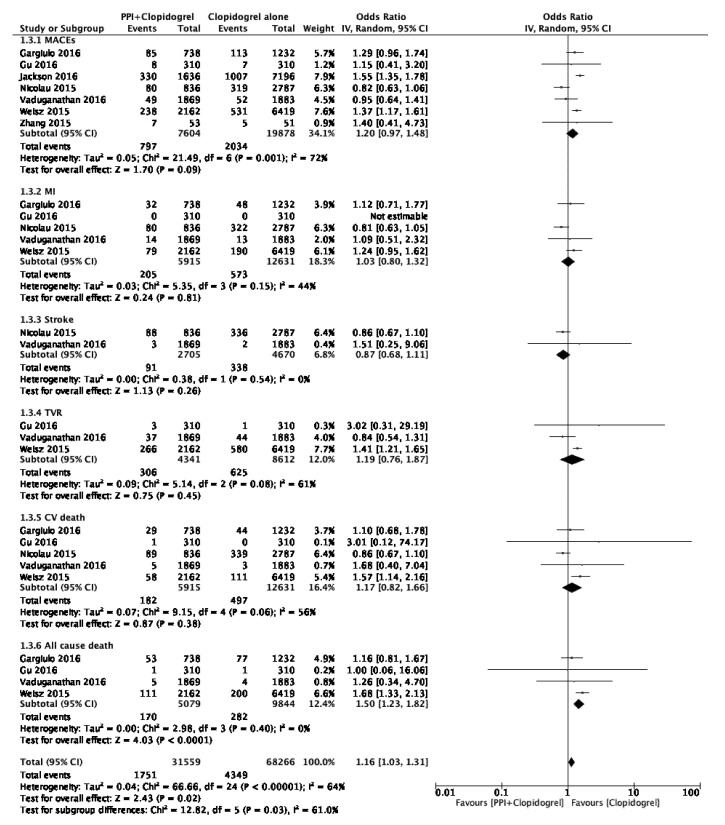
Forest plot illustrating the risk of cardiovascular events associated with the concomitant use of clopidogrel and PPIs using a random-effects model. Zhang 2015 [9]; Gu 2016 [14]; Nicolau 2015 [15]; Gargiulo 2016 [16]; Vaduganathan 2016 [19]; Jackson 2016 [20]; Weisz 2015 [21].

**Figure 5:**
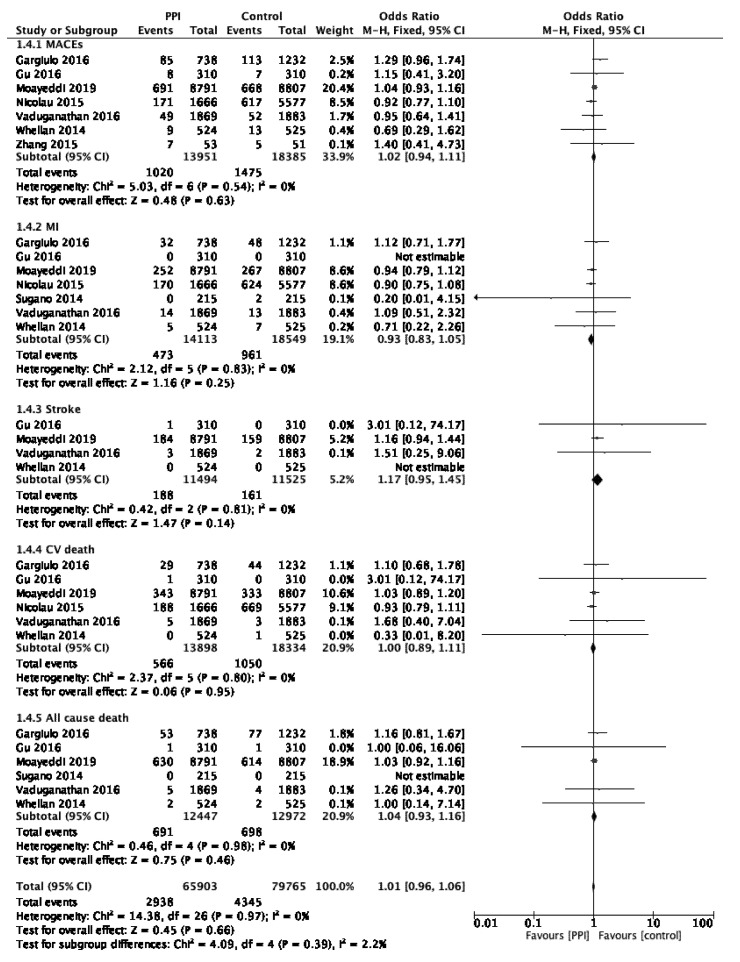
Forest plot illustrating the risk of cardiovascular events associated with the long-term PPI use within the RCTs using a fixed-effects model. Zhang 2015 [9]; Gu 2016 [14]; Nicolau 2015 [15]; Gargiulo 2016 [16]; Moayeddi 2019 [17]; Sugano 2014 [18]; Vaduganathan 2016 [19]; Whellan 2014 [23].

**Figure 6:**
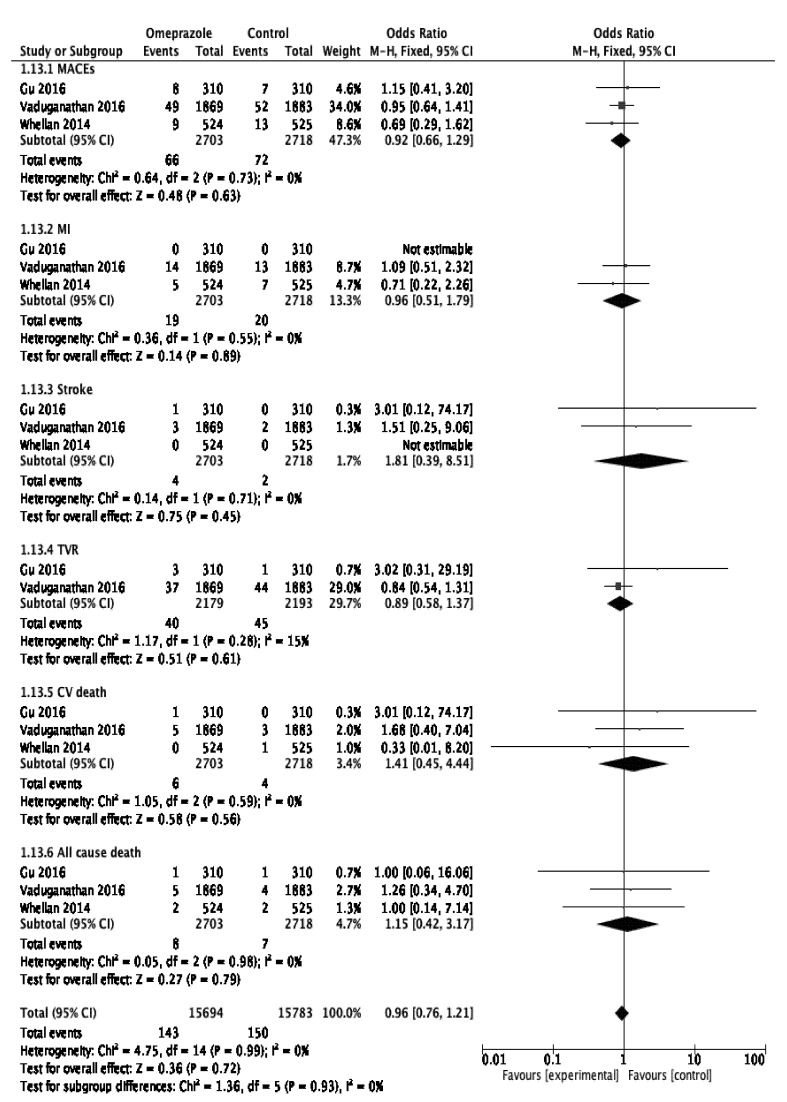
Forest plot illustrating the cardiovascular risk associated with long-term omeprazole use. Gu 2016 [14]; Vaduganathan 2016 [19]; Whellan 2014 [23].

In the first subgroup analysis assessing the cardiovascular risk associated with the concomitant use of clopidogrel and PPIs, we found that this co-therapy heightened the risk of ACD (OR 1.50, 95% CI 1.23–1.82, *p* < 0.001) but did not raise the risks of MACEs (OR 1.20, 95% CI 0.97–1.48, *p* = 0.09), MI (OR 1.03, 95% CI 0.80–1.32, *p* = 0.81), stroke (OR 0.87, 95% CI 0.68–1.11, *p* = 0.26), TVR (OR 1.19, 95% CI 0.76–1.87, *p* = 0.45), or CVD (OR 1.17, 95% CI 0.82–1.66, *p* = 0.38); (Figure 4). However, heterogeneity I^2^ was moderate to high for four outcomes (MACEs, MI, TVR, CVD), ranging from 44% to 72%, and nil for only two outcomes (stroke, ACD). The subgroup analysis of the RCTs (heterogeneity I^2^ = 0% for each evaluated outcome) did not demonstrate any significant association between PPI use and the risks of MACEs (OR 1.02, 95% CI 0.94–1.11, *p* = 0.63), MI (OR 0.93, 95% CI 0.83–1.05, *p* = 0.25), stroke (OR 1.17 95% CI 0.95–1.45, *p* = 0.14), CVD (OR 1.00, 95% CI 0.89–1.11, *p* = 0.95), or ACD (OR 1.04, 95% CI 0.93–1.16, *p* = 0.46) (Figure 5). These results remained stable after excluding the two post hoc analyses [16,17]. The risk of TVR within the RCTs could not be assessed due to a lack of available data. The pulled data from the two included observational studies (OS) demonstrated a significantly increased risk of MACEs in the PPI group (OR 1.42, 95% CI [1.29–1.57], *p* < 0.001; I^2^ = 0%). The other outcomes could not be assessed due to the lack of available data in OS (unpublished figure). Considering the available data within the included studies, the post hoc subgroup analysis regarding specific PPIs only addressed the influence of omeprazole on the outcomes of interest. This pooled analysis, involving three RCTs, found that omeprazole use was not associated with the occurrence of adverse cardiovascular events (overall pooled OR 0.96, CI 95% 0.76–1.21, *p* = 0.72; I^2^ = 0%; Figure 6).

### 3.6. Heterogeneity and Sensitivity Analyses

In order to evaluate the influence of high heterogeneity on the results obtained, we performed sensitivity analyses by excluding each study successively until I^2^ = 0. No sensitivity analyses were performed for the randomized controlled trial subgroup, the observational study subgroup, or the omeprazole subgroup as original heterogeneity I^2^ was nil for these three analyses. PPI use became significantly at risk for TVR in the primary analysis (*p* < 0.001) after excluding one study. All other results remained stable (Table 3). After the exclusion of one to two studies for each outcome, combined PPI/clopidogrel therapy was at increased risk of MACEs (*p* < 0.001), TVR (*p* < 0.001), and CVD (*p* = 0.008). The risk of MI remained insignificant (*p* = 0.29) (Table 3).

### 3.7. Publication Bias

**Figure 7:**
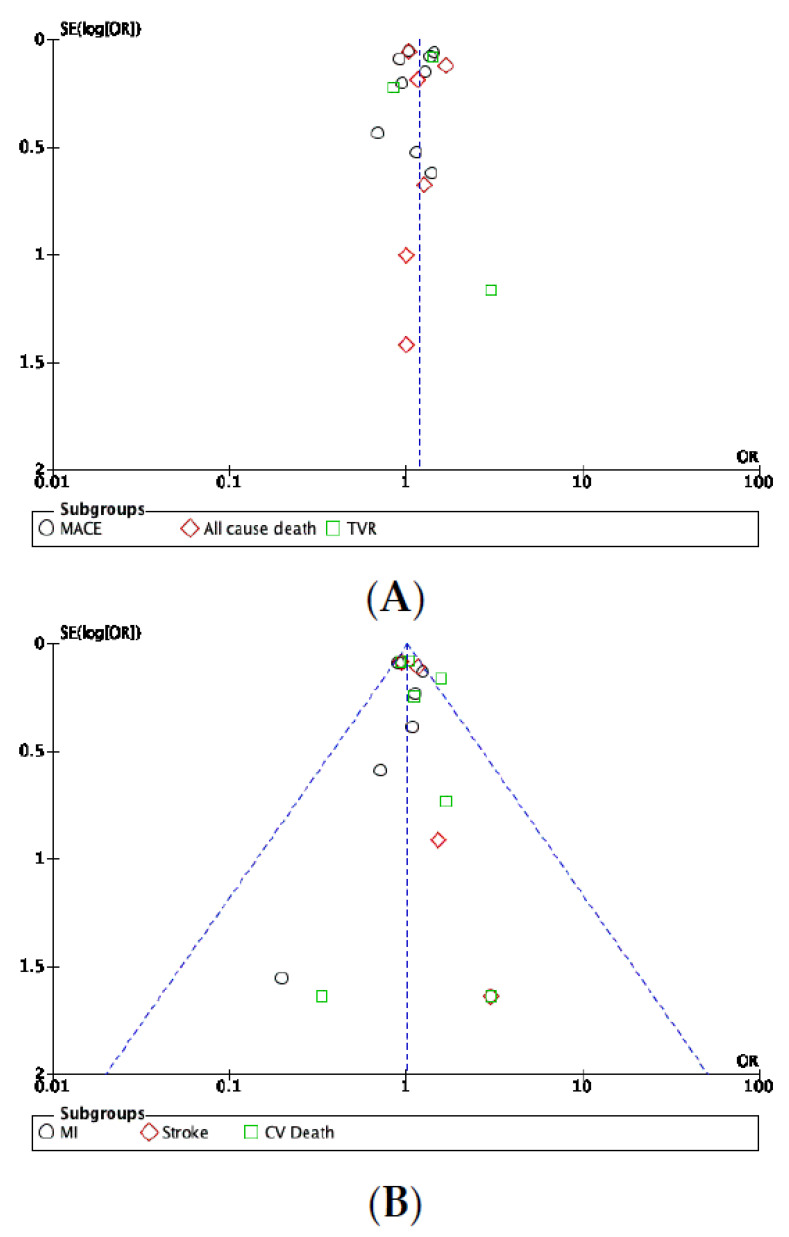

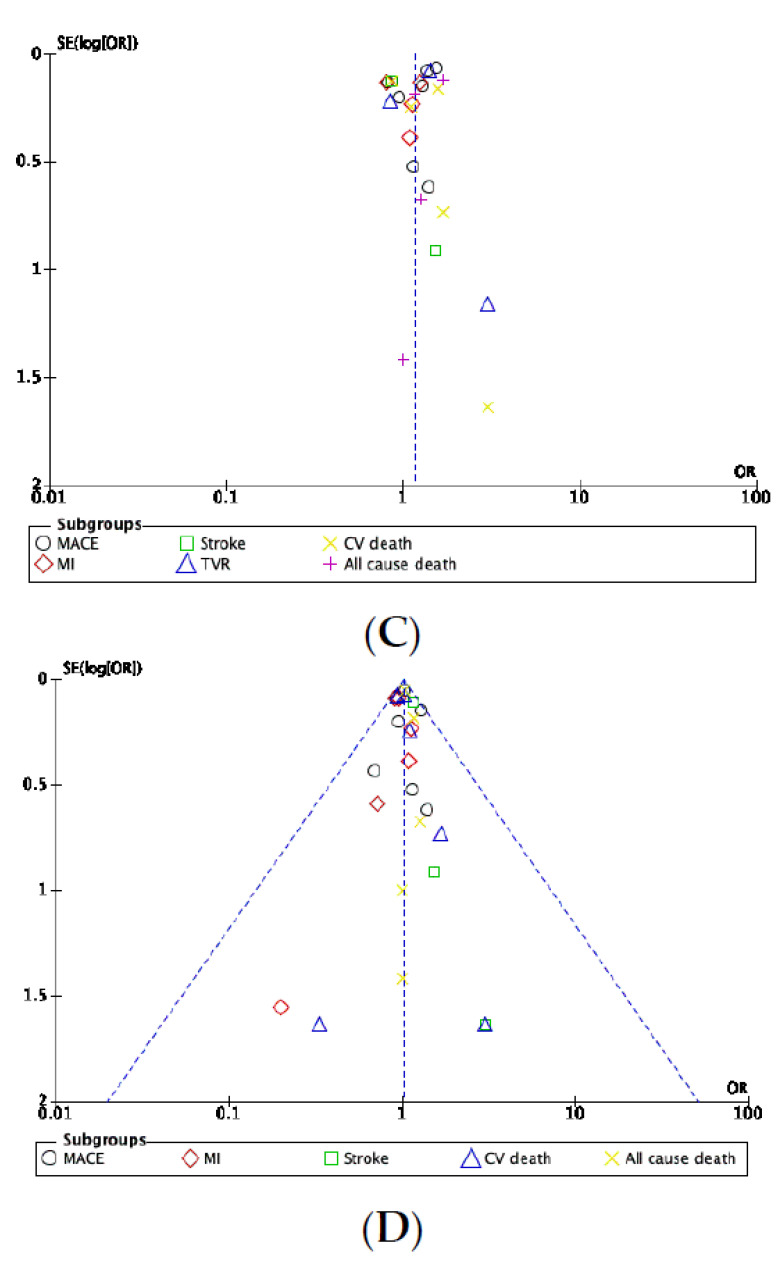
(**A**) Funnel plot of the publication bias within the studies assessed in the primary analysis using a random-effects model. (**B**) Funnel plot of the publication bias within the studies assessed in the primary analysis using a fixed-effects model. (**C**) Funnel plot of the publication bias within studies analyzing the combination of clopidogrel and PPIs. (**D**) Funnel plot of the publication bias within the RCTs.

Visual inspection of the funnel plots performed for the primary analysis and RCT subgroup analysis found no evidence of publication bias (Figure 7A,B,D). Conversely, the funnel plot of the clopidogrel/PPI subgroup analysis suggested the existence of publication bias as the PPI effects estimated in each included study scatter asymmetrically around the summary effect (Figure 7C). The number of studies involved in the omeprazole subgroup analysis was too small to expect relevant interpretation of a funnel plot. Therefore, we did not calculate a funnel plot for this specific analysis.

## 4. Discussion

We conducted this meta-analysis with the aim of assessing the risks of various cardiovascular events associated with long-term PPI use among patients with known atheromatous cardiovascular disease. Overall, the main results of our meta-analysis demonstrated no evidence that PPIs as a drug class were associated with an increased risk of adverse cardiovascular events. However, conflicting results were found for the combined use of PPIs and clopidogrel. Overall, the subgroup analysis involving high I^2^ found that this combined therapy was safe, while the sensitivity analysis that controlled for I^2^ found opposite results. Nevertheless, the PPI/clopidogrel co-therapy subgroup analysis was susceptible to potential publication bias according to the visual interpretation of the funnel plot, which entails a greater risk of publication bias in the sensitivity analysis that included a smaller number of studies. When considering specific PPIs, the independent assessment of omeprazole’s effects on cardiovascular health found it to be safe.

The potential cardiovascular risk associated with PPI use has been studied by several authors, mostly in retrospective observational studies, a study design most likely to lead to selection, confusion, and information bias. It seems that a causal link between PPI exposure and adverse events can hardly be established if there are uncertainties in the measurement of the exposure to PPIs. Moreover, target populations differ from one study to another, which might have resulted in considerable meta-analytic heterogeneity in patients’ baseline characteristics (patients with chronic heart disease +/− acute coronary syndrome +/− post-percutaneous coronary intervention +/− dual antiplatelet therapy including clopidogrel and/or aspirin +/− heart failure; overall population). Several pathophysiological mechanisms have also been put forward to support and justify the study of this risk. However, it must be noted that these articles report conflicting results for both clinical and biological outcomes. Furthermore, we could observe that performing randomized controlled trials versus cohort studies led to diametrically opposite results in most published studies. While cohort studies, prospective or retrospective, tend to support the hypothesis of an increased cardiovascular risk during long-term PPI exposure, randomized controlled trials tend to refute this hypothesis. The same is true for meta-analyses including mostly cohort studies and those including exclusively or almost exclusively randomized controlled trials. Therefore, a significant association between PPI use and cardiovascular events could be more likely related to unmeasured potential confounders than related to a PPI’s proven toxicity.

Finally, most published meta-analyses pulled data from different study designs, which is expected to lead to differences in the observed intervention effects, increasing heterogeneity and weakening the accuracy of the results. [22]

The potential drug–drug interaction between PPIs and clopidogrel that may increase the incidence of cardiovascular ischemic events was the hypothetical case most studied. The increased cardiovascular risk associated with the combined use of clopidogrel and PPIs could result from a competitive interaction between clopidogrel and PPIs with cytochrome P450 isoenzyme 2C19 (CYP2C19), affecting the clopidogrel-specific inhibition of ADP-induced platelet aggregation. Moreover, the conversion of clopidogrel to its active metabolite varied depending on CYP2C19 genetic polymorphisms [27], with 4% to 30% of people being low metabolizers or non-metabolizers, while the others are described as rapid metabolizers [9]. An affinity to CYP2C19 also differed from one PPI to another, with the highest affinity found for omeprazole and the lowest affinity or no affinity found for pantoprazole depending on the study [24,27,28,29,30].

In a meta-analysis of 31 observational studies and 4 RCTs assessing PPI/clopidogrel cardiovascular risk within patients in the post-discharge treatment of unstable angina/non-ST segment elevation myocardial infarction, Melloni et al. [31] found an increased risk of cardiovascular outcomes and stroke in observational studies, while no differences between omeprazole and placebo were found in four RCTs, despite reducing upper gastrointestinal bleeding. Another meta-analysis involving 18 cohort studies (Shi et al. [32]) reflected a higher risk of MACEs and cerebrovascular events (*p* < 0.001), ACD (*p* < 0.001), cardiac death (*p* < 0.001), myocardial infarction (*p* < 0.001), stent thrombosis (*p* < 0.001), TVR (*p* = 0.005), and stroke (*p* = 0.003), with moderate to high I^2^, within patients taking clopidogrel and PPI after stent implantation. In a pooled analysis of 39 studies (31 cohort studies, 8 RCTs and propensity-matched studies), Cardoso et al. [33] found that the concomitant use of PPIs and clopidogrel heightened the risks of ACD (*p* < 0.001), MI (*p* < 0.001), stent thrombosis (*p* = 0.02), acute coronary syndrome (*p* = 0.004), and cerebrovascular accident (*p* < 0.001). Similar results were found in an analysis restricted to cohort studies. However, in a separate pooled analysis of eight RCTs and propensity-matched studies, Cardoso et al. found that combined PPIs and clopidogrel use had no impact on the occurrence of cardiovascular outcomes (ACD *p* = 0.66; ACS *p* = 0.35; MI *p* = 0.65; CVA *p* = 0.34; TVR OR 0.88; *p* = 0.01) while significantly reducing the risk of gastrointestinal bleeding (OR 0.24, *p* = 0.003). After pulling data from 22 cohort studies and 6 RCTs, Lee et al. [34] found that the concomitant use of PPIs and clopidogrel increased the risk of MACEs (*p* < 0.001), CVD (*p* < 0.001), and MI (*p* < 0.001), with high heterogeneity for most analyses up to 90%. Nevertheless, the pulled data from the six RCTs showed no significant association between PPI/clopidogrel co-therapy and the risk of MACEs (*p* = 0.96, I^2^ = 90%). When considering each specific PPI separately in adjusted analyses (I^2^ ranging from 0% to 85%), omeprazole, pantoprazole, and lansoprazole were at increased risk for MACEs, while esomeprazole and rabeprazole were not (*p* = 0.19 and *p* = 0.40, respectively). PPI use was found to be a protective factor against gastrointestinal bleeding (RR = 0.29, *p* < 0.001; I^2^ = 0%). The meta-analysis by Bundhun et al. [35] including nine cohort studies and two RCTs showed that the combination of clopidogrel and PPIs increased the risks of MACEs, MI, stent thrombosis, and TVR but not the risk of mortality for a PPI exposure greater than one year. In a meta-analysis of seven observational studies, Kwok et al. [36] found an elevated risk of MACEs independent of clopidogrel use. Kwok et al. also found an increased risk of MACEs in association with lansoprazole, omeprazole, esomeprazole, and pantoprazole individually when used with clopidogrel.

With regard to biological investigations, Gu et al. [14], Zhang et al. [9], and Lin et al. [37] did not find a significant risk of higher platelet reactivity (*p* = 0.17; *p* > 0.05; *p* = 0.4315 respectively) after measuring platelet reactivity in the blood samples of patients receiving clopidogrel, while Weisz et al. [21] found an opposite result (OR 1.38, 95% CI 1.25–1.52, *p* = 0.001). Sibbing et al. [38] found that omeprazole was significantly associated with a higher platelet aggregation when combined with clopidogrel, while pantoprazole and esomeprazole were not. In relation to CYP2C19 polymorphisms, Furuta et al. [29] reported that omeprazole and rabeprazole significantly lowered the mean inhibition of platelet aggregation (IPA) induced by clopidogrel in rapid metabolizers, while the decreased metabolizers (low and non-metabolizers) were more likely to convert from “responders” (IPA ≥ 30%) to “non-responders” (IPA < 30%) when using a concomitant PPI. They also found that taking PPIs and clopidogrel at two separate times of the day did not prevent the drug–drug interaction between clopidogrel and a PPI. Furuta et al. [29] did not bring to light any difference between omeprazole, rabeprazole, and lansoprazole combined with clopidogrel versus clopidogrel alone regardless of CYP2C19 polymorphisms. In a study enrolling 174 patients, Hokimoto et al. [39] found significantly lower platelet reactivity in patients on clopidogrel and carrying CYP2C19 normal function alleles (extensive metabolizers, EM) compared with patients carrying one (intermediate metabolizers, IM) or two (poor metabolizers, PM) loss-of-function alleles. In line with these results, the cardiovascular event rate was higher in the IM and PM groups than in the EM group. The specific assessment of rabeprazole, a PPI known for having less affinity for CYP2C19, demonstrated no significant differences in residual platelet aggregation or in cardiovascular event rate when combined with clopidogrel versus clopidogrel alone. In a meta-analysis involving four cohort studies and one RCT, Biswas et al. [40] claimed that patients bearing the dual burdens of carrying CYP2C19 loss-of-function alleles and taking PPIs and clopidogrel concomitantly faced a higher risk of major adverse cardiovascular events. However, in studies assessing the influence of CYP2C19 polymorphisms on cardiovascular outcomes, sample sizes appear to be too small for detecting a reliable difference in biological and clinical outcomes.

Independent of clopidogrel use, Dahal et al. [41] demonstrated that PPI use alone was not at increased risk for cardiovascular mortality, all-cause mortality, myocardial infarction, or stroke in a meta-analysis of nine RCTs including patients taking aspirin for the prevention of cardiovascular diseases and stroke. Zhai et al. [42] sought to examine the safety of PPIs for cardiac and vascular health using the Food and Drug Administration Adverse Event Reporting System (FAERS). PPIs were not associated with more cardiac and vascular events compared with the whole database. However, the authors reported a wide range of vascular signals and to a lesser extent cardiac signals. Pantoprazole and esomeprazole showed the broadest spectrums of signals. However, there is no certainty that the reported adverse events are due to the PPIs involved.

Another hypothetical biological mechanism advanced by some authors involves a dysfunction of the vascular endothelium. Endothelial nitric oxide synthase (NOS) is an enzyme that produces the vasoprotective and vasodilator molecule nitric oxide (NO) [43]. Plasma asymmetrical dimethylarginine (ADMA) is an endogenous inhibitor of nitric oxide synthase. Thus, elevated plasma ADMA levels might increase the occurrence of cardiovascular events. In 2013, Ghebremariam et al. [7] published a paper explaining that PPIs elevated plasma ADMA levels by inhibiting an enzyme (dimethylarginine dimethylaminohydrolase) that degrades ADMA. They also found that PPIs reduced nitric oxide levels and endothelium-dependent vasodilatation in a murine model and in ex vivo human tissue. In a cross-over pilot study of 21 adults published in 2015, Ghebremariam et al. [44] found increased plasma ADMA levels in vivo in patients on lansoprazole versus placebo and in patients with a history of cardiovascular disease versus healthy patients. However, these differences were not statistically significant.

### Strengths and Limitations

Our meta-analysis has various strengths. One, unlike most published meta-analyses, we selected studies with evidence levels I or II according to the evidence-based clinical practice guidelines, which aim to reduce methodological heterogeneity as well as ensure data accuracy, internal validity, and relevant results. Two, all included studies were recently published and retrieved from journals of high scientific influence. Three, of the ten studies included, eight were conducted in numerous centers, reinforcing external validity. Four, of the ten included studies, six were randomized controlled trials, of which four were double-blind. Five, the unbalanced distribution of patients’ baseline cardiovascular risk factors did not disserve the main results of our study, as we found an insignificant association between PPI use and the occurrence of adverse cardiovascular events in most conducted analyses. However, heterogeneity within the cardiovascular risk factors could have caused confusion in the sensitivity analysis of the influence of clopidogrel associated with PPIs, which showed evidence of a statistically significant higher risk of cardiovascular outcomes. Our meta-analysis also has several limitations that may have resulted in information, selection, and publication bias. One, the selection criteria that defined the eligible articles favored the quality and the relevance of the included studies but also involved the exclusion of a significant number of references. Two, the number of included studies was small, and the overall heterogeneity between combined studies proved to be substantial. Three, the included studies presented notable differences in terms of patients’ characteristics, follow-up duration, and PPI used. We did not perform adjusted analyses for patients’ baseline characteristics that were not comparable between two groups. Four, in one study [14], the control group took a PPI (pantoprazole), while the control groups in other included studies were given a placebo or called “no PPI”. However, the exclusion of this study from the performed pooled analyses did not change the results obtained. Five, we did not assess the potential dose-related and time-related effects due to the lacks of available data on dosage, frequency, and indication for PPI therapy. Six, we did not corroborate the results of clinical outcomes with the biological mechanisms argued in previous studies. Seven, we did not investigate the potential difference in the rate of gastrointestinal bleeding between PPI use and no-PPI use due to the lack of available data. Eight, the definition of MACEs was different among studies, which may partly explain the high heterogeneity and conflicting results across the analyses we performed for this particular outcome. Nine, we cannot exclude residual confounding variables, mainly in the non-randomized studies, that could affect the comparability between the two groups. Ten, the included post hoc analyses and observational studies collected information on PPI use and outcomes of interest via interviews at baseline and follow-up visits or via medical records, which entails information and confusion bias to varying degrees. Eleven, the visual inspection of the funnel plots alone may lead to the misevaluation of the publication bias of a meta-analysis, especially when combining a small number of studies [33]. Given the aforementioned limitations, the results of this meta-analysis should be taken with caution.

## 5. Conclusions

The overall results of this meta-analysis support the hypothesis that there is no significantly increased risk of cardiovascular events in association with PPI use alone, suggesting that PPIs can be safely used in appropriate clinical settings. The association between the combined use of PPI/clopidogrel and adverse cardiovascular events remained unclear due to substantial bias and inconsistent results across the analyses of the pulled data. These results must be interpreted with caution given the lack of adjustment for known confounders, unmeasured confounders, high heterogeneity, and small number of included studies. Further large-scale randomized controlled trials are required to provide a reliable statement on the safety of PPIs regarding cardiovascular events in association with clopidogrel or not.

## Figures and Tables

**Table 1:** Study characteristics and patients’ characteristics at baseline.

Study, Year	Country	Study Design	Centers	Total Patients (PPI/C)	Follow up Period	Intervention/PPI Type	Control	Outcomes	Age (y)PPI/C	Women (%) PPI/C	Body Mass Index (kg/m^2^) PPI/C	Hypertension (%) PPI/C	Dyslipidemia (%) PPI/C	Diabetes Mellitus (%)PPI/C	History of Smoking (%) PPI/CPPI/C	Clopidogrel (%) PPI/C	Aspirin (%) PPI/C	Prior Myocardial Infarction (%) PPI/C	Prior Stroke (%) PPI/C	CRBT (/7) or NOS (/9)
Zhang et al., 2015 [9]	China	RCT	monocentric	53/51	6 months	Lansoprazole	No PPI	MACEs	64.5/61	55/43	21.9/22.1	51/49	40/39	19/27	40/41	100/100	100/100	NA/NA	NA/NA	4/7
Gu et al., 2016 [14]	China	RCT	monocentric	310/310	6 months	Omeprazole	Pantoprazole	MACEs, CVD, ACD, MI, Stroke, TVR	59.2/58.8	31/29.3	25.6/25.5	65.7/61.2	44.6/41.1	27.7/27	56.1/56.3	>98/>98	>98/>98	15.8/15.1	8.9/8.9	4/7
Nicolau et al., 2015 [15]	Multiple countries	RCT-PHA	multicentric	1666/5577	30 months	Ome, panto, other PPIs	No PPI	MACEs, CVD, MI, Stroke,	63/62	36.5/35.7	NA/NA	80,5/80,3	58,8/59	40.3/38.5	42.9/44.4	50.2/50	93.2/94.2	42.9/44.4	0/0	1/7
Gargiulo et al., 2016 [16]	Italy	RCT-PHA	multicentric	738/1232	2 years	Lanso 90.9%; panto 7.6%; ome, rabe, eso 0.5% each	No PPI	MACEs, CVD,ACD, MI	71.2/68.1	27.5/20.8	26.2/26.9	72.5/71.3	53.8/55.3	23.3/24.8	22.6/24.4	99.9/99.8	100/100	27/26.1	NA/NA	1/7
Moayyedi et al., 2019 [17]	33 countries	RCT	multicentric	8791/8807	3 years	Pantoprazole	Placebo	MACEs, CVD, ACD, MI, Stroke	67.6/67.7	22/21	28.3/28.4	75.9/76.1	88.4/88.8	38/38	66.3/66.1	NA/NA	NA/NA	61.5/61	4/4	7/7
Sugano et al., 2014 [18]	Japan, Korea, Taiwan	RCT	multicentric	215/215	72 weeks	Esomeprazole	Placebo	ACD, MI	66.1/68.1	19/21	NA/NA	NA/NA	NA/NA	NA/NA	NA/NA	0.5/2	100/100	66.5 */69.2 *	66.5 */69.2 *	7/7
Vaduganathan et al., 2016 [19]	Multiple countries	RCT	multicentric	1869/1883	6 months	Omeprazole	Placebo	MACEs, CVD, ACD MI, Stroke, TVR	65.9/65.9	33.2/30.6	29.5/29.5	79.7/81	78.8/76.8	31.6/28.5	13.6/15.1	64.6/64.6	100/100	30.2/28.2	7.2/8	6.5/7
Whellan et al., 2014 [23]	USA	RCT	multicentric	524/525	6 months	Omeprazole	No PPI	CVD, ACD, MI,MACEs, Stroke	66.3/65.7	28.4/28.8	31/31.1	NA/NA	NA/NA	NA/NA	NA/NA	21.2/21	100/100	40.8/37.9	19.5/21.5	7/7
Jackson et al., 2016 [20]	USA	OS	multicentric	2167/9788	12 months	PPIs	No PPI	MACEs	63/59	34.1/26.6	30/29	76.1/64.8	73,1/63.9	32.4/25.2	NA/NA	75/74	97.9/98.3	24.5/18.4	7.7/4.9	7/9
Weisz et al., 2015 [21]	USA, Germany	OS	multicentric	2162/6419	2 years	PPIs	No PPI	CVD, ACD,MACEs, MI, TVR	64.4/63.2	29.9/24.1	29.5/29.5	83.7/77.8	76.9/73.2	34.8/31.4	22.7/22.6	100/100	100/100	28.6/23.7	NA/NA	8/9

CRBT, Cochrane risk-of-bias tool; Nos, Newcastle–Ottawa scale; PHA, post hoc analysis; RCT, randomized controlled trial; OS, observational study; PPIs, proton pump inhibitors; H2RA, histamine H2 receptor antagonists; NA, not available; MI, myocardial infarction; CVD, cardiovascular death; ACD, all-cause death; MACEs, major adverse cardiovascular events; TVR, target vessel revascularization; C, Control; lanso, lansoprazole; ome, omeprazole; panto, pantorazole; rabe, rabeprazole; eso, esomeprazole. * History of cardiovascular events requiring aspirin in secondary prevention.

**Table 2:** Newcastle–Ottawa scale scores for the observational studies.

Study	Selection (0 to 4 *)	Comparability (0 to 2 *)	Outcome (0 to 3 *)	Total	Quality Assessment
Jackson	3 *	2 *	2 *	7 *	Good
Weisz	3 *	2 *	3 *	8 *	Good

*: stars awarded for each numbered item within the Selection, Comparabilty and Outcome categories according to the Newcastle-Ottawa quality assessment form for cohort studies [26].

**Table 3:** Sensitivity analysis summary (I^2^ = 0%).

Meta-Analysis	Outcome	Removed Studies	Results
Primary Analysis	MACEs	[20,21]	OR 1.02, 95% CI 0.94–1.11, *p* = 0.62
	TVR	[19]	OR 1.42, 95% CI 1.22–1.65, *p* < 0.01
	CVD	[21]	OR 1.00, 95% CI 0.89–1.11, *p* = 0.95
	ACD	[21]	OR 1.04, 95% CI 0.93–1.16, *p* = 0.45
Clopidogrel Analysis	MACEs	[15,19]	OR 1.45, 95% CI 1.31–1.60, *p* < 0.001
	MI	[21]	OR 0.89, 95% CI 0.72–1.11, *p* = 0.29
	TVR	[19]	OR 1.42, 95% CI 1.22–1.65, *p* < 0.001
	CVD	[15]	OR 1.42, 95% CI 1.09–1.84, *p* = 0.008

MACEs, major adverse cardiovascular events; MI, myocardial infarction; TVR, target vessel revascularization; CVD, cardiovascular death; ACD, all cause death.

## Supplementary Materials

The following supporting information can be downloaded at: https://www.mdpi.com/article/10.3390/jcm11144096/s1, Table S1: Results of literature research for each database.
