# Supplementary material for: The Safety of Long-Term Proton Pump Inhibitor Use on Cardiovascular Health: A Meta-Analysis
Source: J Clin Med. 2022 Jul 15;11(14):4096. doi: 10.3390/jcm11144096 (PMC9322047; doi:10.3390/jcm11144096)
Supplement: Supplementary file 1 [file jcm-11-04096-s001.zip › jcm-1824281-supplementary.pdf]

Supplementary Table S1: Results of literature research for each database

Search	Query	Results		
		**Pubmed**	**Embase**	**Cochrane**
#4	**Search: ((#1) AND (#2)) AND (#3) Filters: from 2014/1/1 - 2022/4/30**	820	676	221
#3	**Search: ((adverse effect) OR (adverse drug reaction)) OR (risk)**	4,730, 897	3,651,675	380,972
#2	**Search: (((cardiovascular disease) OR (anti-platelet therapy)) OR (clopidogrel)) OR (aspirin)**	2,848,335	331,311	67,700
#1	**Search: ((proton pump inhibitor) OR (proton pump inhibitors)) OR (PPIs)**	31,165	33,013	4441
